# Spiral CT angiography in an infant with a hypoplastic aortic arch

**DOI:** 10.2349/biij.2.2.e11

**Published:** 2006-04-01

**Authors:** S Man Harun, Y Faridah

**Affiliations:** Department of Biomedical Imaging, Faculty of Medicine, University of Malaya, Kuala Lumpur, Malaysia

## Abstract

The advent of multislice computed tomography (CT) has revolutionised the performance of body CT and allowed the development of CT angiography (CTA). CTA is a robust and minimally invasive method of visualizing the arterial vascular system. The newer generation of multidetector scans has allowed for shorter scanning times with no respiratory misregistration at peak vascular opacification following peripheral contrast injection. The volume of data obtained from these scans can be further manipulated to generate two-dimensional (2D) and three-dimensional (3D) images with no increase in radiation to the patient. Hence, CTA has gained popularity and importance as the alternative diagnostic tool especially for ill patients in which conventional angiography is inadvisable.

We present an infant with coarctation of the aorta and hypoplastic aortic arch, in which CT angiography was used to pinpoint the diagnosis. The CT findings were subsequently confirmed at surgery.

## CASE REPORT

A full term newborn was noted to have poor Apgar score at birth. Physical examination revealed differential cyanosis with maximal upper limb and lower limb saturation difference of more than 10%. The child also had a heart murmur with absent femoral pulses bilaterally. On day two of life, the child developed respiratory distress and was admitted to the intensive care unit for ventilation.

Chest x-ray demonstrated cardiomegaly with congested lung fields. Echocardiogram suggested the presence of a hypoplastic transverse arch. She also had a moderate sized muscular ventricular septal defect with bi-directional shunting. As the patient was ill, CT angiography with 3D reconstruction was performed rather than a conventional angiography.

Spiral CT imaging was carried out using GE Lightspeed 16-slice multidetector CT (GE Healthcare, Milwaukee, Wisconsin, USA). A bolus application of 5 mL non-ionic contrast (l.5 mL/kg body weight) followed by 5 mL of bolus saline was hand-injected via a peripheral venous cannula. Scanning commenced after 50% of contrast volume was injected. Spiral CT of the aortic arch and aorta was performed, starting at the level of the thoracic inlet vessels down to the mid abdomen in 1.25 mm slice thickness. KV was set at 120 while mA was set at 220. Total scan time was 12 seconds, during which the ventilation was withheld. Axial slices were reconstructed with a 60% overlap (0.75 mm / l.25 mm). Subsequently, 2D and 3D reconstructions from various angles were produced with standard software.

**Figure 1:**
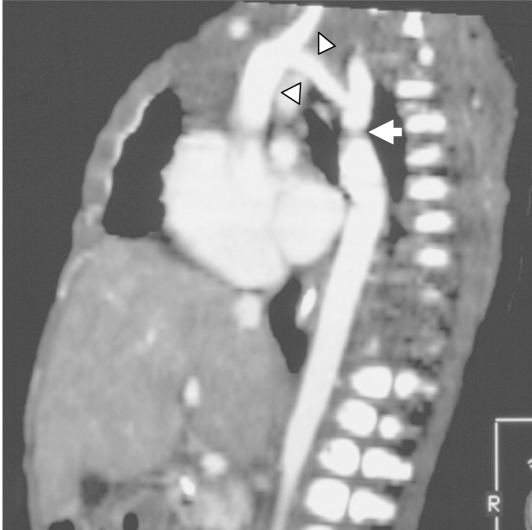
2D reconstruction showing hypoplastic aortic arch (arrowheads) with coarctation of aorta (arrow).

**Figure 2:**
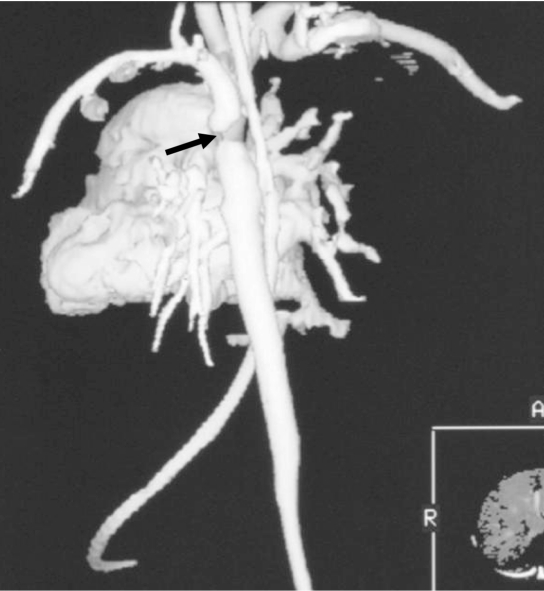
3D reconstruction displays the coarctation of the aorta (arrow).

Spiral CT showed that the arch was hypoplastic (Figure 1). The left subclavian and the left internal thoracic arteries arise from the descending aorta distal to the hypoplastic arch. The left common carotid artery arises from the left subclavian artery. Immediately distal to the origin of the left subclavian artery, there is a stenosis of the descending thoracic aorta in keeping with post-ductal coarctation (Figure 2). The intercostals arteries are well seen arising from the descending thoracic aorta distal to the coarctation. There is cardiac failure as the hepatic veins are dilated and the lungs appear congested. The pulmonary artery is large and patent, and the pulmonary veins drain normally into the left atrium.

On day 14 of life the child underwent open-heart surgery and the CT findings were confirmed. It was noted that the degree of hypoplasia of the transverse arch was severe and would have caused difficulty in cardiac catheterisation, a traditional method of diagnosing this condition.

## DISCUSSION

Coarctation of the aorta with concomitant hypoplastic aortic arch is a rare congenital anomaly affecting the cardiovascular system [2]. Traditionally, diagnosis is made at cardiac catheterisation [3]. Cardiac catheterisation is an invasive procedure and therefore is fraught with complications especially when performed in ill infants. Furthermore, the severity of the hypoplastic arch could cause difficulty in cardiac catheterisation as was noted intraoperatively in this patient.

An exciting alternative such as CT angiography with 3D reconstruction may provide similar if not superior findings as conventional angiography. There is an added advantage in that the surrounding structures are also delineated on CT scan. The spectrum of indications for CT examination has expanded since the introduction of the spiral CT technique, especially with regards to vascular abnormalities. This is mainly attributed to faster scanners and the ability to perform 2D and 3D reconstructions without additional radiation to the patient. High quality 2D reconstructed images are generated from transaxial scans provided that image data was acquired with a thin collimation and transaxial images were generated with a high degree of overlap [4]. In addition, Maximum Intensity Projection (MIP) reconstructions can be rendered in any projection, including projections impossible to obtain with conventional angiography [5]. MIP is a volume-rendering technique in which parallel rays pass through the volume of data and the maximum CT number encountered in each ray is displayed [1]. Therefore MIP permits separation of the enhanced lumen from the surrounding structures.

In comparison with angiography, where several injections of contrast media are necessary, CT angiography can be performed following single bolus injection of contrast media and in this case only a total of 5 mL was used. The disadvantages of CTA such as side effects of contrast media could be disregarded in comparison to cardiac catheterisation, which uses a higher volume of contrast.

The main limiting factor of CT examination would be the radiation dose. In this patient, the estimated effective dose of the patient is in the region of 10.6 mSv for CT imaging of chest to the mid abdomen [6]. A conventional diagnostic cardiac angiography would generally produce median effective dose of about 4.6 mSv for a paediatric patient [7]. However the effective dose during cardiac catheterisation increases in newborn with effective doses ranging from 6.5 mSv at 50th percentile to as high as 18.0 mSv at 90th percentile [8]. Therefore, the radiation dose of CTA in newborns is comparable to that of conventional cardiac catheterisation. In this case, the CTA gave invaluable information regarding the patient’s diagnosis and subsequent treatment. Furthermore, the grave condition of the patient necessitates the non-invasive approach of CT.

Other techniques such as ultrasound and MRI have its advantages in cardiac imaging. However, 2D echocardiography is operator dependent and as this is a rare case, an exact diagnosis was difficult to achieve. MRI was not performed due to the long examination time and the need for arrested respiration to achieve images of diagnostic quality, which is unsuitable due to the unstable condition of the patient.

Thus, CT angiography has become a promising procedure for the visualisation of vascular disorders. It has a shorter examination time, and a reduced number of projections and amount of contrast media are needed compared to cardiac catheterisation. Its radiation dose is comparable to that of conventional angiography in the newborn. In addition, CTA is helpful for pre-operative planning as the localisation, shape and length of the stenosis, as well as the course of the collateral vessels, can be assessed clearly.

## CONCLUSION

In conclusion, spiral CT angiography is proven to be a reliable method in demonstrating anomalies of the aorta in a paediatric patient. It is also a practical clinical test that may be an alternative to cardiac catheterisation, especially in delineating extracardiac vascular structures.

## APPENDIX

Two movie clips showing 3D reconstructed CT angiography of the procedure performed for this case report is available for download at: http://www.biij.org/2006/2/e11
